# Supplementary material for: Chromothripsis during telomere crisis is independent of NHEJ, and consistent with a replicative origin
Source: Genome Res. 2019 May;29(5):737–49. doi: 10.1101/gr.240705.118 (PMC6499312; doi:10.1101/gr.240705.118)
Supplement: Supplemental Material [file supp_gr.240705.118_Supplemental_file_1.zip › contigs/annotated_contigs/DB53/contig.2.DB53_length_466_mean_cov_7.8991416309.docx]

**DB53_length_466_mean_cov_7.8991416309**

```
GCTCAATTAGAAAGCGACATACAAGATAAACACAA|GGTCCAAAAAACAAGACAGGGAGACCATTAGCATAGCTATTATTAAGTGTTGC
                                    >chr8:135549215-135549542 + E=2e-179
ACAGAAGTTCTTAACCAAAGTAATGAGATCTAACATGATAAATATTACTGATTTCTCTAATTTTTACTGATAGCTACTATATGCCAGAT

AGCTCTAAATGTTTTGCATGGATTAATTCCTATAAGTCTCACATCAAGCTCATATGGTACGTGCAATTATTATCCTCATTTTTACAGGT

AAGGAAACTGAGACACAGAATGGTTTAGAGACTTGCCATAGTTTCACAGTCTCCCAGCCTGGTTACTTGGTTTGCATCAGATTTGGGGC

CCCAG|GC|ATTACACACTGATGTACACCTCCATGGTGCACCCGCCTCCATCACACACTGATGTGTGCCTCCATGGTGCACTGCCTGCA
      >chr7:155828495-155828601 + E=3e-52
TCACATACTGATCTTGCAGGCCAC
```
